# Clinical research in oncology: in memory of Professor Gordon McVie

**DOI:** 10.3332/ecancer.2022.1340

**Published:** 2022-01-13

**Authors:** Ian F Tannock

**Affiliations:** Princess Margaret Cancer Centre, 610 University Avenue, Toronto ON M5G 2M9, Canada

**Keywords:** problems in drug development, clinical trials, pharmacology

## Abstract

Gordon McVie campaigned throughout his career for merging scientific and clinical expertise and for investigating the underlying pharmacokinetics and pharmacodynamics in clinical trials. This need remains highly relevant today when most cancer clinical trials investigate agents that target a known molecular pathway, yet anticancer drug development has changed minimally from that used for chemotherapy when more was better and substantial toxicity inevitable. Here, I summarise some common problems that confound current drug development, including problems in interpreting results of phase 3 randomised trials, as well as trials investigating personalised medicine.

Memories of wandering the halls at the European Society for Medical Oncology (ESMO) or other major cancer meeting, and suddenly I hear that unmistakeable Scottish voice, ‘*Ian – let’s grab a coffee – there’s something important I want to talk to you about*’. Larger than life and bursting with passion, there would be Gordon McVie – and who could or would want to refuse such an invitation? I might not always agree with him but conversations with Gordon were never mundane. When I heard of his death, it took a while to stop asking myself what bolt of lightning could strike down this seemingly indestructible person.

Gordon McVie and I have pursued careers in medical oncology over similar time frames – from the relatively early days of chemotherapy to the present where most new drugs are designed to interact with a known biological target, and thereby to inhibit the survival or proliferation of cancer cells. Chemotherapy developed from empirical observations that certain chemicals were toxic to dividing cells; the war gas sulphur mustard begat the anticancer drug nitrogen mustard, which in turn begat a family of alkylating agents including cyclophosphamide. Greater scientific knowledge led to the development of antimetabolites, including methotrexate, whose pharmacology was studied by Gordon and his colleagues [1], but almost all chemotherapy drugs acted against dividing cells in general rather than cancer cells in particular. More recent drug development has depended on increasingly sophisticated knowledge about the genetics and epigenetics of cancer, about how the protein products of genes control tumour cell proliferation and survival, and about the tumour microenvironment and interactions between tumour cells and immune cells. One of many contributions from Gordon McVie was his early recognition that advances in cancer treatment would depend on merging the two solitudes of laboratory and clinical research – which he championed tirelessly in his several roles, but particularly when he led the UK’s Cancer Research Campaign (CRC).

In 1984, Gordon McVie published an article entitled ‘The Role of Pharmacokinetics in (Combination) Chemotherapy’ [2] and the opening sentence stated: ‘*It seems inconceivable that a new drug could reach widescale application without thorough understanding of its pharmacology’*. In 1987, he co-authored a report of a workshop hosted by the European Organization for Research and Treatment of Cancer, of which he later became President, on ‘Problems and Prospects in Current Therapeutic Application of Molecular Biology’ [3]. The final sentence of this report stated: ‘*The dialogue between clinicians and laboratory scientists in molecular biology must increase if possibilities of controlling cancer by novel and selective means are going to be translated into reality’.* Moving on some 35 years it is pertinent to ask if these lessons have been learnt, and whether the situation has improved in the current era of targeted anticancer medications. I think the answer is yes, but only partially!

During the period when new anticancer drugs were chemotherapeutic agents, a paradigm for clinical research was established. Phase 1 trials asked what is the maximal tolerated dose (MTD) in people with cancer for whom known beneficial treatment was no longer available, and what are the toxicities? It was assumed that more was better, and that patients would tolerate inevitable toxicity. Assays for drugs and their metabolites had generally been established, but rarely was there an attempt to use such information to optimise schedule, or for antimetabolites to demonstrate the extent and duration of target inhibition. The classical 3+3 design was established where small groups of patients received increasing doses until a limit was set by toxicity. Phase 2 trials evaluated antitumour effects (usually with the endpoint of response rate) in patients with a given type of cancer treated with the MTD, sometimes using signals of benefit from preclinical studies of cell lines or from the occasional response seen in phase 1 studies to identify which types of tumour might respond. And if there was reasonable evidence of anti-tumour activity, randomised phase 3 trials would compare the new drug with prior standard treatment, either alone or in combination, again with dose guided by early studies.

So, what has changed in the current age of targeted anticancer therapy? Unfortunately, not much. The optimal dose and schedule for such agents is that which provides and maintains effective inhibition of the molecular target. Yet most phase 1 trials have not changed: most of them are still designed to determine the MTD, and for many agents this exceeds substantially the minimum effective dose needed to inhibit the molecular target, and only occasionally is target inhibition evaluated in early phase trials. There are many examples of targeted anticancer drugs which have been registered at high doses despite lack of evidence of a dose–response relationship in early trials, or in spite of evidence that lower doses provide similar target inhibition [4–6]. One example includes the androgen synthesis inhibitor, abiraterone acetate, which is highly effective in improving survival and its quality at several stages of progression of metastatic prostate cancer. A quarter of the approved dose of 1,000 mg, given with food rather than fasting, was shown to have similar pharmacokinetic and pharmacodynamic effects, and similar therapeutic effects using a proximal endpoint in a small, randomised trial that recruited 72 men with advanced prostate cancer [7]. With colleagues, I applied successfully to have this lower dose recognised as an acceptable alternative by the National Comprehensive Cancer Network, and adoption of the lower dose can dramatically lower cost and improve access to this effective therapy in lower- and middle-income countries (LMICs) [8]. Other examples include the anti-Programmed Cell Death Protein 1 (anti-PD1) immunotherapy drugs pembrolizumab and nivolumab. These agents are highly effective in improving survival for people with several types of cancer including melanoma, kidney, non-small cell lung cancer and others, but there is minimal access in LMICs due to their high price. Yet there is substantial evidence for their efficacy at markedly lower doses and less frequent schedules than those approved by the United States Food and Drug Administration (FDA) and the European Medicines Association [9–12]. Trials are finally underway to evaluate more affordable schedules.

While on-target effects might occur at substantially lower doses, off target effects contributing to toxicity are dose related, including financial toxicity. A reason for the non-scientific ‘more is better’ approach is that it is easier and faster for a sponsoring company to undertake a classical series of phase 1 and 2 trials than to undertake dose optimising studies, and the sooner a drug can be brought to market, the higher the profit for the sponsoring company. In general, the registration agencies, the FDA and EMA, have not required studies to optimise dose and schedule, although there are encouraging recent signs that this might change with the FDA requiring a comparison of the MTD with a lower dose for the new drug sotorasib, an inhibitor of a specific *KRAS* mutation [13].

And what about the status of clinical research in generating modern phase 3 clinical trials? There are several types of scientific expertise that need to come together to optimise such investigations: (i) science related to the underlying mechanisms by which the targeted agent under investigation might improve the outcome of patients, including the optimal dose and schedule to inhibit the target, but also evaluation of biomarkers that identify the population most likely to benefit and (ii) the science underlying the optimal design of clinical trials to ensure the validity of their results, their freedom from bias and uncertainty and their relevance to patients seen in everyday practice. Research into predictive biomarkers has improved, allowing the appropriate selection of patients for some types of treatment, evaluation of Human Epidermal Growth Factor Receptor 2 (HER2) expression in breast and gastric cancer and Epidermal Growth Factor Receptor (EGFR) and Anaplastic Lymphoma Kinase (ALK) mutations in Non-Small Cell Lung Cancer (NSCLC) being examples that enable selection of treatments that have little or no effect in patients lacking these biomarkers. But there is substantial room for improvement: for example, responses to immune checkpoint agents relate (imperfectly) to expression levels of Programmed Cell Death Ligand 1 (PD-L1) in tumour cells or infiltrating immune cells in several types of cancer. Yet at least three different methods of assessing such expression are in use, and companies sponsoring clinical trials have managed to expand approval for their drugs by lowering the level of PD-L1 expression to the lowest value above which patients appear to benefit, despite knowledge that benefit is actually limited to a smaller group with a higher cut-off [14]. We need to lobby the registration agencies to prevent such disingenuous practice.

Although many high quality, practice-changing phase 3 trials have been completed and published, there remains considerable potential to improve the design, analysis and reporting of others [15, 16]. The expense of mounting such trials has increased substantially so that now almost 90% are sponsored by pharmaceutical companies; these trials generally have very stringent entry requirements with exclusion of older patients and those with any co-morbidity [17]. The goals of benefitting patients and of generating a profit are sometimes, but not always, congruent and it is essential that oncologists review the results of such trials with a constructive but critical eye, as doubtless Gordon McVie would have taught his mentees. Ideally, trials need to have wider entry criteria so that they are relevant to patients seen in everyday practice; platform trials such as STAMPEDE for prostate cancer represent an excellent example for admitting all patients who might benefit from treatment, as well as early dropping of arms that show no signs of benefit and adding new arms as interesting questions emerge from other data [18]. Some of the problems that have led to hype and inflated claims of benefit for randomised phase 3 trials are listed in the Box.

**Box:** Some problems in the design, analysis and reporting of phase 3 randomised clinical trials [15–17]

Inappropriate choice of control group

Use of a primary endpoint that does not reflect benefit to patients

Failure to recognise informative censoring as a source of bias if Progression-Free Survival is used as the endpoint

No or inappropriate measure of Quality of Life in palliative trials

Use of a secondary endpoint or subgroup analysis to draw conclusions

Under-reporting of toxicity

Dependence on a *p*-value to indicate a ‘positive’ trial rather than effect size or a measure of clinical benefit

Use of spin and bias to inflate apparent benefit in reporting results

Many reports are drafted by medical writers employed by or contracted to the sponsor

Strict entry criteria and failure to recognise the efficacy–effectiveness gap

Benefit is greater and toxicity less in clinical trials compared to a real-world population

Finally, a few words about personalised or precision medicine, which many would regard as the apotheosis of the merging of scientific and clinical expertise as proposed long ago by Gordon McVie while instituting polices as director of the CRC. The success of treating groups of patients with a driving mutation by using an appropriately targeted drug, such as imatinib for chronic myelogenous leukaemia and trastuzumab for HER2-positive breast cancer led to the reasonable assumption of 1–2 decades ago that if we could determine driver mutations in any individual cancer, and target the mutation with an appropriate drug, we would have similar success. That hypothesis has since been tested in multiple basket trials (where patients with different tissue origins of cancer but with a common driver mutation are treated with an agent targeting the mutation) or umbrella trials (where patients with a given type of cancer undergo genetic sequencing and are treated with different drugs targeting different mutations). More than 20,000 patients have been included in published trials and unfortunately this experience has been disappointing [19, 20]. Of patients screened for entry into such trials, only about 3% eventually respond to a selected targeted agent, but such trials continue despite the need to reassess and recognise the huge complexity that limits this approach, and in particular the heterogeneity in space and time of molecular pathways, and their Darwinian selection, that allow progression of human cancers [21, 22]. We need to inform our patients that there is no unique genetic sequence of their tumour, and scientists and clinicians need to reprogramme their research to allow for the molecular complexities of tumours.

## Funding

No funds were used to produce this article.

## Conflicts of interest

The author has received reimbursement from Bayer, Janssen and Roche-Genentech for time served as a member or chair of Data Safety Monitoring Committees for pharmaceutical sponsored clinical trials, but otherwise does not accept payments from commercial enterprises.
